# Influence of Vitamin D-Related Gene Polymorphisms (*CYP27B* and *VDR*) on the Response to Interferon/Ribavirin Therapy in Chronic Hepatitis C

**DOI:** 10.1371/journal.pone.0074764

**Published:** 2013-09-20

**Authors:** Elena García-Martín, José A. G. Agúndez, María L. Maestro, Avelina Suárez, Marta Vidaurreta, Carmen Martínez, Cristina Fernández-Pérez, Luis Ortega, José M. Ladero

**Affiliations:** 1 Department of Biochemistry and Molecular Biology, University of Extremadura, Cáceres, Spain; 2 Department of Pharmacology, University of Extremadura, Cáceres, Spain; 3 Genomics Unit, Clinical Laboratory Department, Hospital Clínico San Carlos, Instituto de Investigación Sanitaria del Hospital Clínico San Carlos (IdISSC), Madrid, Spain; 4 Service of Clinical Microbiology, Hospital Clínico San Carlos, Instituto de Investigación Sanitaria del Hospital Clínico San Carlos (IdISSC), Madrid, Spain; 5 Clinical Research and Methodology Unit. Hospital Clínico San Carlos, Medical School, Universidad Complutense, Instituto de Investigación Sanitaria del Hospital Clínico San Carlos (IdISSC), Madrid, Spain; 6 Service of Pathology, Hospital Clínico San Carlos, Instituto de Investigación Sanitaria del Hospital Clínico San Carlos (IdISSC), Madrid, Spain; 7 Service of Gastroenterology, Hospital Clínico San Carlos, Department of Medicine, Medical School, Universidad Complutense, Instituto de Investigación Sanitaria del Hospital Clínico San Carlos (IdISSC), Madrid, Spain; University of Navarra School of Medicine and Center for Applied Medical Research (CIMA), Spain

## Abstract

**Background and Aims:**

Vitamin D exerts immunomodulatory effects on the host response against infection with hepatitis C virus (HCV). This study was performed to assess the putative influence of polymorphisms in vitamin D-related genes on the response to antiviral therapy in patients with chronic hepatitis C (CHC).

**Methods:**

Single nucleotide polymorphisms (SNPs) in *CYP27B*-1260 gene promoter (rs10877012AC) and in vitamin D receptor (*VDR*) gene rs2228570TC, rs1544410CT, rs7975232AC and rs731236AT were analyzed in a cohort of 238 Caucasian CHC patients treated with pegylated interferon (Peg-IFN) plus ribavirin (RBV). Multivariate analyses were performed to exclude confounding effects of well-known baseline predictors of response to therapy (HCV genotype and load, *IL28B* genotype, age, and GGT and serum cholesterol).

**Results:**

Three SNPs at the *VDR* gene (rs1544410, rs7975232 and rs731236) were in strong linkage disequilibrium, with the CCA haplotype predicting therapeutic failure [Odds ratio 2.743; (95% C.I. 1.313–5.731), p = 0.007]. The carrier state of the *VDR* rs2228570 T allele was inversely related to the probability of therapeutic failure [Odds ratio 0.438; 95 C.I. (0.204–0.882), p = 0.021]. No relation existed between *CYP27B*-1260 rs10877012 polymorphism and response to therapy. The area under the operating curve (AUROC) based on the model including all variables significantly related to the response to therapy was 0.846 (95% confidence interval = 0.793–0.899).

**Conclusion:**

*VDR* gene polymorphisms are independently related to the response to Peg-IFN+RBV therapy in chronic hepatitis C and could be used as complementary biomarkers of response when included in a prediction algorithm in association with demographic, virologic, biochemical and genetic traits.

## Introduction

The success rate of current therapy for chronic hepatitis C (CHC) that combines pegylated interferon plus ribavirin is related to a variety of predictive factors that can be used as response biomarkers. Some of these depend on the hepatitis C virus (HCV), such as viral genotype, changes in critical regions of the viral genome and viral load. Other are host-related, either genetic (*IL28B* gene polymorphisms, gender and race) or acquired (insulin resistance, obesity, liver steatosis, iron overload and liver fibrosis stage) [1], [2]. Hence, by combining the most reliable of these biomarkers at baseline [3] it is possible to make an approximate prediction of the probability of achieving sustained virologic response (SVR, defined as non-detectable HCV RNA in serum 6 months after the end of therapy). The adjunction of new direct anti-HCV drugs is changing the therapeutic approach in CHC, but at present a still significant number of CHC patients are treated with the classic two-drug approach. To refine the predictive capacity biomarkers of response to pegylated interferon plus ribavirin additional baseline predictors of clinical response would be welcome.

Calcitriol - 1,25(OH)_2_ Vitamin D - is a component of the hormonal system that maintains calcium and phosphorus homeostasis. In addition, calcitriol shows immunomodulatory effects as it reduces the levels of proinflammatory cytokines and promotes innate immune response. Calcitriol is synthesized in many tissues and may act as a ubiquitous immune regulating factor [4]–[6].

Vitamin D deficiency is very common in patients with CHC [7]–[9]. This deficiency may reduce the rate of success of interferon plus ribavirin combined antiviral therapy [10]–[11], although two recent reports [12], [13] have challenged the existence of this relation. The biological effects of calcitriol depend on its rate of synthesis, which is catalyzed by several enzymes involved in the 1–25 double hydroxylation of calciferol. Cytochrome P-450 CYP27B1 generates 1–25 (OH)_2_ vitamin D, mainly in the kidney. Active Vitamin D must interact with its specific transmembrane receptor (VDR) to exert its physiological functions [6]. Both *CYP27B1* and *VDR* genes have polymorphisms which possibly influence the efficacy of antiviral therapy, as it has recently been suggested by two independent groups [14]–[16]. In a previous study we did not identify any immediate effect of isolated vitamin D therapy on HCV viral load, although we did confirm that vitamin D deficiency is very common in Spanish CHC patients [17], but at a similar level to that found in the general Spanish population [18]. In the present study we aim to evaluate polymorphisms in Vitamin D-related genes as baseline biomarkers of response to interferon-ribavirin therapy in patients with CHC.

## Patients and Methods

### Patients

The population study included all patients with CHC who were scheduled to receive combined double therapy with pegylated interferon and ribavirin in accordance with current guidelines [19], and who attended since June 2003 to the outpatient clinic of the Gastroenterology Department (Liver Unit), San Carlos University Hospital, Madrid, Spain, Only patients who had completed a full course of therapy or who had stopped therapy due to therapeutic failure, defined with widely accepted criteria [19], were included in the study. Patients who had had to stop therapy due to drug intolerance, and those co-infected with HIV or HBV or with superimposed hepatocellular carcinoma were not included. The study was designed in accordance with the guidelines of the Declaration of Helsinki and approved by the ethics committee of the San Carlos University Hospital, Madrid, Spain. Written informed consent was obtained for all participants. The final study group was composed of 238 patients of Caucasian descent (221 Spaniards). Baseline and clinical characteristics are summarized in Table 1. Blood samples for biochemical, virologic and genetic studies were obtained during the month previous to the onset of therapy. Histological data were collected when a liver biopsy performed within the previous six months was available. In the remaining cases the results of transient elastography were included, when available.

**Table 1:** Baseline characteristics of the 238 patients with chronic hepatitis C.

Variable^(1)^	All patients	Sustained virological response	Failure of therapy	Statistics (Univariate)
Gender				
Male, n(%)	148 (62.2)	57 (62.0)	91 (62.3)	Odss ratio (95% C.I.) = 0.984(0.575–1.685)
Female, n(%)	90 (37.8)	35 (38.0)	55 (37.7)	
Age (*years*)				
Mean (range)	49.9 (20–77)	46.6 (20–74)	52.0 (24–77)	1.04 (1.01–1.07). p<0.001
HCV genotype, n (%)				
1	208 (87.4)	73	135	Chi^2^ = 13.92 (p = 0.003)
* 1b*	*157 (66.0)*	*53*	*104*	
* 1non b*	*51 (21.4)*	*20*	*31*	
2	2 (0.8)	2	0	
3	14 (5.9)	11	3	
4	13 (5.5)	6	7	
n.a.^2^	1 (0.4)	0	1	
Genotypes 1–4/genotypes 2–3	221/16	79/13	142/3	Odss ratio (95% C.I.) = 7.789(2.144–28.16)
HCV RNA				
<400.000 IU/ml, n (%)	46 (19.2)	27	19	Odss ratio (95% C.I.) = 2.798(1.447–5.410)
≥400.000 IU/ml (%)	190 (79.8)	64	126	
n.a.^2^	2 (0.8)	1	1	
Fibrosis stage (METAVIR), n (%)^3^				
0	20 (8.4)	10	10	Chi^2^ = 8.46 (p = 0.076)
1	61 (25.6)	24	37	
2	35 (14.7)	13	22	
3	53 (22.3)	17	36	
4	33 (13.9	5	28	
n.a.^2^	36 (15.1)	23	13	
Necroinflammatory grade (METAVIR), n (%)				
1	19 (8.0)	10	9	Chi^2^ = 2.89 (p = 0.236)
2	67 (28.2)	21	46	
3	77 (32.4)	28	49	
n.a.^2^	75 (31.5)	33	42	
Steatosis, n (%)				
No	103 (43.4)	37	66	Odds ratio (95% C.I.) = 1.189(0.618–2.289)
Yes	60 (25.1)	24	36	
n.a.^2^	75 (31.5)	31	44	
ALT, U/L	104 (75)	108 (69)	100 (79)	0.999 (0.995–1.002). p = 0.134
GGT, U/L	89 (95)	62 (63)	146 (105)	1.008 (1.003–1.013). p<0.001
Cholesterol, mg/dL	176 (35)	185 (36)	171 (32)	0.998 (0.980–0.996). p = 0.006
*IL28B* rs12979860 genotype n (%)				
CC	79 (33.2)	54	25	Chi^2^ = 43.56 (p<0.001)
CT	124 (52.1)	31	93	
TT	34 (14.3)	7	27	
n.a.^2^	1 (0.4)	0	1	
T allele non carriers/T allele carriers	79/158	54/38	25/120	Odds ratio (95% C.I.) = 6.82(3.75–12.41)

1 Continuous variables are given as mean (SD) except age.

2 n.a.: Data not available.

3 The stage of fibrosis was established by liver biopsy in 163 cases and by transient elastography in 39 cases.

Subset analyses were performed in patients infected with viral genotype 1.

### Laboratory Methods

Routine hematological and biochemical tests were performed with the standard methods at our laboratory.

#### HCV analysis

Quantitative analysis of HCV-RNA was performed with the Cobas Amplicor HCV Monitor version 2.0 (Roche Molecular Diagnostic, Madrid, Spain). The detection range was 600 IU/mL to 8.5×10^5^ IU/mL. Starting from July 2005, viral RNA was extracted automatically using Cobas AmpliPrep, and the viral load was detected using Real-Time polymerase chain reaction (PCR) with Cobas TaqMan (Roche Diagnostics, Madrid, Spain) which has a detection range of between 15 IU/mL and 2×10^8^ IU/mL [20]. Viral load was classified as low (<400.000 IU/ml) or high (≥400.000 IU/ml), according to Witthöft *et al*. [21].

The Hepatitis C viral genome is highly variable and is classified into 6 genotypes groups, or clades, based on phylogenetic analyses of the genomic sequence. HCV genotypes were determined by a reverse hybridization assay (VERSANT HCV Genotype 2.0 Assay (LiPA); Siemens, Tarrytown, NY USA). Biotinilated DNA PCR product generated by RT-PCR amplification of the 5′UTR and core region of the HCV RNA is hybridized to immobilized oligonucleotide probes. The probes, which are bound to a nitrocellulose strip by a poli(dT) tail, are specific for the 5′UTR and core region of different genotypes. The VERSANT HCV Genotype 2.0 Assay (LiPA) is a line probe assay, for in vitro diagnostic use, which identifies Hepatitis C Virus genotypes 1 to 6 and subtypes a and b of the genotype 1 in human serum or EDTA plasma samples.

Chen et al. [22] communicate that the accuracy of 1a and 1b subtyping ranges from 80% to 95% by 5′UTR analysis, depending on the specific isolated tested, so the VERSANT HCV Genotype 2.0 Assay (LiPA) uses sequence motifs from the core region in addition to the 5′UTR to improve the accuracy of the identification of 1a and 1b.

Liver biopsy specimens were scored using the METAVIR system [23]. All biopsies were evaluated and scored by the same pathologist.

#### Analyses of Vitamin D-related genes

Genotyping was carried out by the use of TaqMan probes designed to detect the following SNPs: *CYP2B7B1,* rs10877012 (custom TaqMan SNP assay; Applied Biosystems, Madrid, Spain); *VDR*, rs2228570, rs731236, rs1544410 and rs7975232 (TaqMan SNP assays C__12060045_20, C__2404008_10, C___8716062_10 and C__28977635_10, respectively; Applied Biosystems, Madrid, Spain). The SNPs were selected on the basis of allele frequencies and functional of clinical implications [12]–[14]. The only nonsynonymous SNP confirmed for the *VDR* gene was included in the study. No other nonsynonymous SNPs have been described to occur in Caucasian individuals with minor allele frequencies over 0.001 (see the website http://www.ncbi.nlm.nih.gov/projects/SNP/snpref.cgi?showRare=on&chooseRs=coding&go=Go&locusId=7421).

For every SNP analysed, twenty samples with heterozygous genotypes and up to twenty samples with homozygous genotypes (homozygous non-mutated and homozygous mutated when available), were determined as blind duplicates by direct sequencing of the amplified fragments. In all cases the genotypes fully corresponded to those obtained with TaqMan probes. As a prerequisite, only individuals with full genotyping were scheduled to be included in the study. All possible haplotypes combining the four VDR SNPs analyzed were constructed and their frequencies were analyzed by using PHASE as described elsewhere [24]. Putative departures of Hardy-Weinberg Equilibrium and SNP linkages were calculated by using the software Haploview 4.1. In addition, the rs12979860 *IL28B* gene polymorphism was determined in 236 patients as described previously [25].

### Statistical Analysis

A database was created to include clinical and demographical data, and baseline biochemical, virologic, histological and genomic results.

Statistical analysis was performed using SPSS 17.0 (SPSS Inc, Chicago, Il, USA). Therapy response was divided into two categories: responder patients obtaining SVR and non-responder patients. This latter category including patients with primary failure, partial viral response and relapse after end-of-therapy viral response. Statistical associations were calculated by univariate analysis comparing the two groups defined according to therapy response for age, sex, viral genotype (classified as difficult- (1 and 4) *vs.* easy-to-treat (2 and 3)), viral load (low *vs.* high), serum ALT, GGT, and cholesterol and the studied polymorphisms at *CYP27B, VDR* and *IL28B* genes. Continuous variables, expressed as mean (SD), were compared with the Student’ *t* test or the Mann-Whitney *U* test, each when adequate, depending on their Gaussian distribution. A p value <0.05 was considered significant. Categorical variables were compared with the χ^2^ or the Fisher exact tests, each when appropriate, and the effect of differences was established by calculating the odds ratio with the 95% confidence interval.

The variables different from a p value <0.05 in the univariate analysis were included in a multivariate analysis based on a logistic regression model by exact methods (maximum likelihood tests) to identify which were independently related to the result of therapy. *CYP27B* and *VDR* gene polymorphisms were forced into the multivariate analysis except when strong linkage disequilibrium was detected between *VDR* SNPs (see below).

## Results

Three of the four SNPs at the *VDR* gene (rs1544410, rs7975232, and rs731236) were in strong linkage disequilibrium and hence patients were categorized as non carriers/carriers of the allelic combination rs1544410C - rs7975232C - rs731236A, defined as the risk haplotype in agreement with previously published evidence [14]. The remaining SNP at the *VDR* gene (rs 2228570 TC) was not in linkage disequilibrium with the others and was included in the analysis as an independent variable.

In the univariate analysis the following variables were significantly linked with therapeutic failure: older age, *IL28B* rs12979870T allele carrier state, higher viral load, viral genotype 1 or 4, higher GGT levels and lower serum cholesterol levels. These variables were included in the multivariate analysis along with the *CYP27B* and *VDR* SNPs and the risk haplotype. In the multivariate analysis, all variables previously identified in the univariate analysis were confirmed as predictors of therapy failure at a significance level equal to p<0.05, with the exception of advanced age, as was, in addition, the carrier state of the of the risk CCA haplotype (combined rs1544410C - rs7975232C - rs731236A alleles) (Odds ratio = 2.743 - 95% confidence interval = 1.313–5.731- p = 0.007). On the contrary, the carrier state of the minor T allele at *VDR* rs2228570 was significantly related to the probability of obtaining sustained viral response to therapy Odds ratio = 0.438 −95% confidence interval = 0.204–0.882- p = 0.021 (Table 2).

**Table 2:** Logistic regression analysis of demographic, biochemical and virological variables at baseline and of *CYP27B1, VDR* and *IL28B* gene polymorphisms in relation with the lack of response (failure to antiviral therapy) in 238 patients with chronic hepatitis C.

Variable (1)	Result of therapy		Statistical analysis	
	Sustained virological response	Primary failure	Univariate(2)	Multivariate(3)
Age	46.6 (10.4)	52.5 (9.4)	1.04 (1.01–1.07).	1.031(0.995–1.068). *p = 0.133*
CYP27B_rs10877012 T allele carrier (no/yes)	47/45	57/39	0.654(0.386–1.111)	0.650(0.327–1.290). *p = 0.218*
VDR_rs2228570 T allele carrier (no/yes)	33/60	38/58	0.640(0.375–1.096)	**0.438** **(0.204–0.882). ** ***p = 0.021***
Carrier of rs1544410 BsmI C/rs7975232 ApaI C/rs731236 TaqI A VDR allelic combination (no/yes)	66/27	64/32	1.498(0.856–2.620)	**2.743** **(1.313–5.731). ** ***p = 0.007***
*IL28B* rs12979860 T allele carrier (no/yes)	54/39	9/84	6.82(3.75–12.41)	**8.724** **(4.031–18.88). ** ***p<0.001***
Viral load (low/high/unknown)	28/64/1	9/87/0	2.632(1.348–5.137)	**4.286** **(1.392–9.507). ** ***p<0.001***
Viral genotype 1/non-1	74/20	91/5	0.129(0.036–0.464	**6.268** **(1.365–28.79). ** ***p = 0.018***
GGT	61 (61)	125 (118)	1.008(1.003–1.013)	**1.006** **(1.001–1.011). ** ***p = 0.025***
Cholesterol	185 (37)	167 (32)	0.998(0.980–0.996)	**0.982** **(0.971–0.992). ** ***p = 0.001***

(1) Continuous variables are given as mean (SD).

(2) Odds ratio (95% confidence interval).

(3) Odds ratio (95% confidence interval) and *p value* in all significant variables in the univariate analysis. *CYP27B1*and *VDR* gene polymorphisms were forced into the analysis.

The probability of therapeutic failure (P) was estimated with the formula:
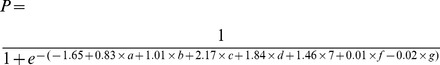
where the substitution values were as follow: **a** : *VDR* rs10877012T: non carrier = 1; carrier = 0; **b** : *VDR* rs1544410BsmIC/rs7975232ApaIC/rs731236TaqIA haplotype: non carrier = 0; carrier = 1; **c** : *IL28B*rs1297860T: non carrier = 0; carrier = 1; **d** : Viral genotype: 1 or 4 = 1; 2 or 3 = 0; **e** : Viral loal: <400.000 IU/mL = 0; ≥400.000 IU/mL = 1; **f** : GGT (U/L); g : Serum cholesterol (mg/dL).

**Figure 1:**
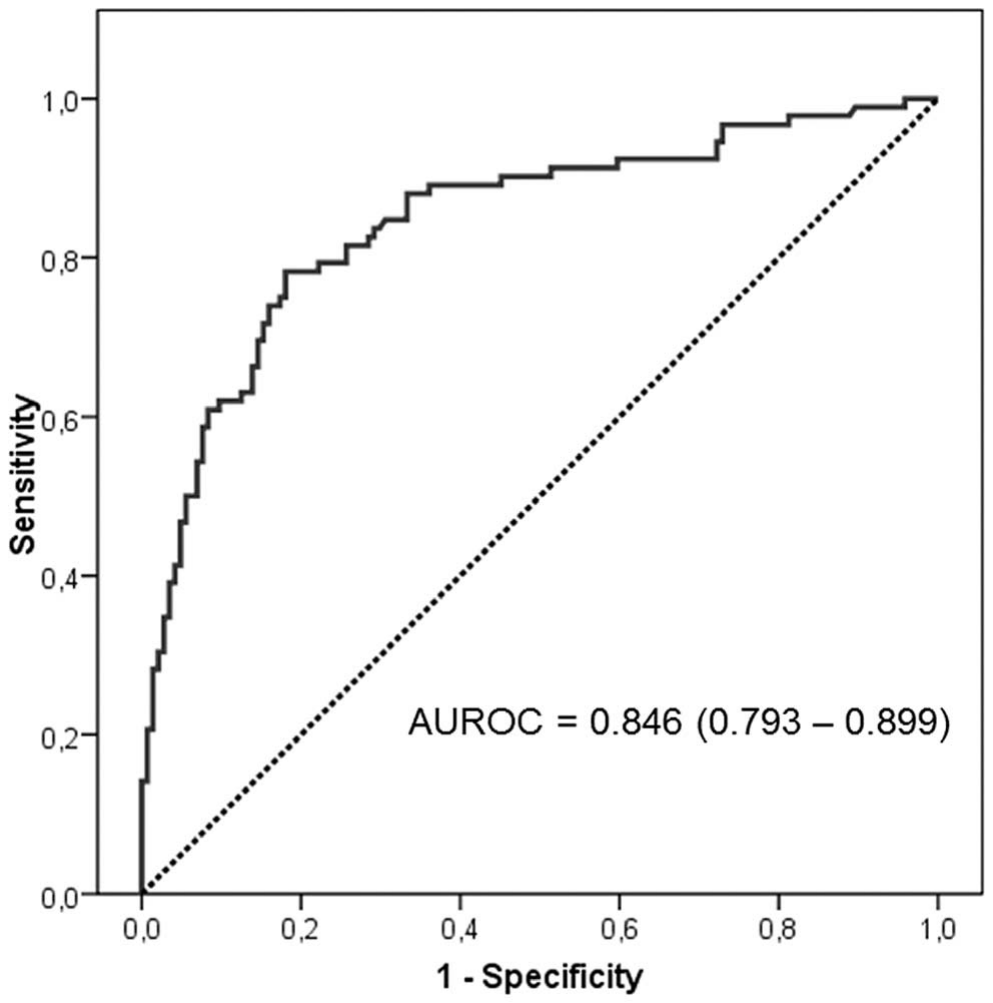
Receiver-operating curve provided by the model constructed to establish the predictive value for therapeutic failure. Area under the receiver-operating curve = 0.846 (95% confidence interval = 0.793–0.899).

The receiving operating curve (ROC) was plotted in accordance with the same model to establish specificity and sensitivity values (Figure 1). The best cut-off value for this model was 0.620 that provided a sensitivity of 78.3% and a specificity of 79.2% for predicting therapeutic failure.

In the analysis restricted to the 208 patients infected with viral genotype 1 the differences detected in the whole series remained significant in the multivariate analysis although at a lower level, both for the carrier state of the *VDR* rs2228570 T allele (Odds ratio for SVR = 0.469 (0.223–0.990). *p = 0.042* and for the *VDR* CCA haplotype (Odds ratio for therapy failure = 2.179 (1.000–4.762). *p = 0.044*).

## Discussion

This study reveals that a common nonsynonymous SNP in the *VDR* gene (rs2228570 T/C), which is studied here for the first time in chronic hepatitis C patients, is a predictor of the clinical outcome of combined interferon plus ribavirin therapy. This polymorphism causes a threonine-metionine change in the Vitamin D receptor and, in the multivariate analysis that included the most potent predictors of viral response to combined therapy at baseline, those patients carrying the minor T allele in homo- or heterozygosis obtained SVR at a higher rate than patients with the rs2228570 CC genotype (p = 0.015). Most of these patients were infected with difficult-to-treat viral genotypes (87.4% genotype 1 and 5.5% genotype 4). When the analysis was restricted to the subset of genotype 1 infected patients, these differences remained significant.

In addition, we also analyzed three highly linked SNPs in the *VDR* gene previously studied by Baur et al. in 155 Swiss patients with chronic hepatitis C [12]. Only 62.9% of their patients were infected with genotype 1. These authors reported a significant association (p = 0.028) between failure of the combined therapy and the haplotype bAt. In our study we found that these three SNPs are in strong linkage disequilibrium (95%), leading us to categorize our patients as non carriers/carriers of the rs1544410C - rs7975232C - rs731236A allelic combination. Eighty three patients carrying this allelic assortment had a highly significant lower rate of SVR in multivariate analysis (Pc = 0.009).

Vitamin D 1α-hydroxylase, the *CYP27B1* gene product, catalyzes the synthesis of 1–25 (OH)_2_ Vitamin D, the active form of vitamin D that binds to the vitamin D receptor. Its immediate precursor, 25(OH) Vitamin D, is considered to be an adequate marker of vitamin D state [6], but not necessarily of vitamin D physiologic activity because a genetic-induced reduction of 1–25(OH) vitamin D synthesis may result in a functional deficit of vitamin D, as occurs in vitamin D-dependent rickets type I. However, in a recent study, the *CYP27B1*-1260 promoter polymorphism (rs10877012) also included in our study is not related with vitamin deficiency in children [26].

The retrospective design of this study precluded us to determine serum vitamin D and hence to analyze the possible influence of the studied polymorphisms on serum concentrations of the vitamin. Available data on the *CYP27B1*-1260 promoter polymorphism (rs10877012) are contradictory, as Lange et al. suggested that this polymorphism could influence serum concentrations of 1–25(OH)_2_ vitamin D [15] whereas Kitanaka et al. [26] did not confirm such a relationship. However, these authors reported that the *Bsm*I, *Apa*I, *Taq*I haplotype at the *VDR* gene (that we have designed as CCA haplotype) is found 5.61 times more frequently among Vitamin-D deficient Japanese children than in the control group. None of the Vitamin D-related polymorphisms included in our study was found to be related with serum vitamin D levels in a recent GWAS analysis [27].

Lange et al. in a complementary study based on a greater series of CHC patients (701 cases), detected an association between *CYP27B1*-1260 promoter polymorphism (rs10877012) and the rate of response to antiviral therapy (p = 0.06) which reached significance (p = 0.02) when the analysis was limited to patients with the *IL28B* genotype associated with poor response [16]. In the present study we have not confirmed such association although the sample size of our study group was only a third of that included by Lange et al. A sample size of 370 cases (α <0.05; power >80%) would have been necessary to exclude unit from the 95% confidence interval of the observed odds ratio (1,539), which is similar to that found by Lange et al [16].

Advanced liver fibrosis negatively influences the probability of response to antiviral combined therapy in chronic hepatitis C [27]. In the present study a liver biopsy was available in 163 (68.5%) patients, with data provided by transient elastography in other 39 patients (see table 1). Data on the stage of fibrosis were lacking in 36 patients and this kept us to include this variable in the multivariate analysis aimed to detect the possible influence of the studied vitamin D-related polymorphisms on the results of antiviral therapy. However, we found that the relative frequencies of the studied polymorphisms were quite similar in the group of patients classified according to their fibrosis stage as null-low (F0–F1) *vs.* moderate-advanced (F2–F4). (Data not shown).

In this study, data on insulin resistance, that is a known predicting factor of therapeutic failure, are lacking. Prospective studies taking into consideration all these factors are warranted.

We conclude that two polymorphic sites in the *VDR* gene that influence the response rate to interferon-ribavirin therapy in chronic hepatitis C exist. The association of these polymorphisms with the response rate is independent from other well known predictors of response at baseline that were included in the multivariate analysis as potentially confounding factors (*IL28B* gene polymorphism, viral genotype and load, age, and serum GGT and cholesterol levels). In addition to the mechanistic implications of alteration in VDR with regard to clinical response to interferon-ribavirin therapy in chronic hepatitis C, which deserves further studies, the use of these new criteria may add to the refinement in the prediction of clinical response based on ancillary baseline data.
